# Genetics in Genomic Era

**DOI:** 10.1155/2015/364960

**Published:** 2015-03-25

**Authors:** Eugenia Poliakov, David N. Cooper, Elena I. Stepchenkova, Igor B. Rogozin

**Affiliations:** ^1^National Eye Institute, NIH, Bethesda, MD, USA; ^2^Institute of Medical Genetics, School of Medicine, Cardiff University, Cardiff, UK; ^3^Saint Petersburg Branch of N.I. Vavilov Institute of General Genetics, Russian Academy of Sciences, Saint Petersburg, Russia; ^4^National Center for Biotechnology Information, NIH, Bethesda, MD, USA

Genetics is a relatively young science compared to many other fields in biology, for example, evolutionary biology or physiology. However, genetics is a central theme in many fields, especially the medical sciences, because genetic studies can provide explanations and may even allow predictions to be made, in the context of a range of biological problems including the field of inherited human disorders. This has become especially important during the last decade as we have entered the “genomic era.” With the dramatic improvement of sequencing technologies and the enormous reduction in the cost of sequencing, biologists are faced with a “data avalanche.” Next generation whole exome or genome sequencing has provided us with an arsenal of tools to study human, animal, and plant genetics. The underlying genetic lesions responsible for Mendelian diseases may now be found and mapped with even a limited number (as few as one) of affected individuals (rare and neglected diseases), with the help of large quantities of “control” genome data such as those emanating from the 1000 Genomes Project. Genomic data analysis may also be useful for the dissection of the genetic mechanisms underlying complex polygenic diseases or in exploring the role of modifiers genes in influencing the age of onset or clinical severity of a given Mendelian disease entity. Although we have acquired new research capabilities, we are also encountering new problems with the analysis of genomic data and the reanalysis of already published (deposited in databases) data. Such problems of genomic data presentation, format, sharing, and reanalysis are now starting to be addressed.

This special issue is dedicated to problems of genetics in the genomic era and comprises five articles: three review articles and two research articles. In the first review article, entitled “Unlimited Thirst for Genome Sequencing, Data Interpretation, and Database Usage in Genomic Era: The Road towards Fast-Track Crop Plant Improvement,” A. P. Dhanapal and M. Govindaraj discuss the use of crop plant databases in advancing research in the genomic era. The major focus of this review is to provide knowledge on platforms for comparative genomics of agriculturally important crop plants with industrial and environmental significance. Recent advances in sequencing and resequencing of plant genomes have potentiated new analyses of genomic variation and gene function. Genetic variation databases could facilitate research that helps to improve the efficiency of plant breeding programs. This review should aid researchers in the plant science research community by providing information on available databases and platforms for genome-based analyses that help to link model systems with other plants in the genomics context. This is a timely review which highlights the recent finding of frequent gene and whole genome duplication events. Many unresolved questions however remain regarding the number and timing of such events in plant evolution.

Gene duplication is a key mechanism of genomic change in evolution. I. B. Rogozin discusses recent analyses of the “ortholog conjecture” (OC) hypothesis. Under the OC hypothesis, which is central to the functional annotation of genomes, orthologous genes are functionally more similar than paralogous genes at the same level of sequence divergence. A recent study found a greater functional similarity, in terms of gene ontology (GO) annotations and expression profiles, among within-species paralogs as compared to orthologs. These findings have suggested that the functional similarity of homologous genes is primarily determined by the cellular context of those genes, rather than their evolutionary history. Subsequent studies have suggested that the OC hypothesis appears to be generally valid but that a comprehensive picture of the evolution of gene expression requires the incorporation of lineage-specific aspects of paralogy. The observed complexity of gene expression evolution after duplication may be most parsimoniously explained by the duplication-degeneration-complementation model combined with selection for gene dosage.

E. Poliakov et al.'s research paper reinforces the central importance of gene expression. Some ninety years on from the first articulation of the Warburg theory of cancer cell origin, the question of altered metabolism in cancer is again assuming a central role. Analyses of signaling pathways and oncogenes in different types of cancer have been the focus of research for several decades. Now, empowered by a wealth of knowledge about tumor suppressor genes, oncogenes, and signaling pathways, the reprogramming of cellular metabolism (e.g., increased glycolysis to respiration ratio in cancer cells) has reemerged as a key step in cancer progression. To obtain a general picture of cancer metabolism and to analyze the level of expression of various genes encoding proteins including metabolic enzymes across various cancers, E. Poliakov et al. employed dbEST and Unigene data. With the appearance of abundant RNA-seq cancer data, it is interesting to ascertain if dbEST-based conclusions will hold. The authors delineated a list of genes that are overexpressed in most types of cancer. They also grouped overexpressed enzymes into KEGG pathways and analyzed adjacent pathways to describe the enzymatic reactions that take place in cancer cells thereby identifying major players in the cancer protein machinery. Glycolysis/gluconeogenesis, oxidative phosphorylation, and pyruvate metabolism appear to be the most abundant pathways although several other pathways are enriched in genes from the list. Ubiquitously overexpressed genes could be marked as nonspecific cancer-associated genes when analyzing genes that are overexpressed in certain types of cancer. Thus, the list of overexpressed genes is likely to be a useful tool for systems biology approaches to cancer research.

D. Hmida-Ben Brahim et al. discuss Huntington disease (HD) (an autosomal dominant neurodegenerative disorder). The causative mutation is an expansion of more than 36 CAG repeats in the first exon of the* HTT* (IT15) gene. Many studies have shown that the* HTT* gene interacts with several modifier genes to regulate the age at onset of HD. Their study aims to investigate the involvement of CAG expansion and 9 modifiers in the age at onset variance of 15 HD Tunisian patients. The authors establish a correlation between these modifier genes and the age of onset of this disease. Their results demonstrate a specific effect of modifier genes in each population. Despite the small number of studied patients, this report constitutes the first North African study of Huntington disease patients.

This special issue is concluded by an article by M. Srinivasan et al. that discusses the importance of genetic diversity assessment in crop plants and its recent advances. The importance of plant genetic diversity is now being widely recognized by agricultural scientists. This paper comprehensively reviews four important areas: (i) the significance of plant genetic diversity (PGD); (ii) the risk associated with the narrowing down of the genetic base of current commercial cultivars and climate change; (iii) analysis of existing PGD analytical methods in both the pregenomic and genomic eras; and (iv) the tools now available for PGD analysis in the postgenomic era. This review describes the new methods and technology for the improved and rapid assessment of the genetic diversity of crops and for the utilization of germplasm from gene-banks in their applied breeding programs. Since plant breeding research and cultivar development are integral components of improving food production, the availability of and access to diverse genetic stocks will help make the global food production network become more sustainable. The pros and cons of the use of basic and advanced statistical tools available for measuring genetic diversity are discussed.

We have no doubt that there are many topics that remain uncovered in this special issue. However, we hope that the approaches described here will become widely used by the scientific community.

*Eugenia Poliakov* *Eugenia Poliakov* *David N. Cooper* *David N. Cooper* *Elena I. Stepchenkova* *Elena I. Stepchenkova* *Igor B. Rogozin* *Igor B. Rogozin*

